# Lump, multi-lump, cross kinky-lump and manifold periodic-soliton solutions for the (2+1)-D Calogero–Bogoyavlenskii–Schiff equation

**DOI:** 10.1016/j.heliyon.2020.e03701

**Published:** 2020-04-15

**Authors:** Harun-Or- Roshid, Mahbub Hassan Khan, Abdul-Majid Wazwaz

**Affiliations:** a Department of Mathematics, Pabna University of Science and Technology, Pabna, 6600, Bangladesh; b Department of CSE, Pundra University of Science and Technology, Bogra, Bangladesh; c Department of Mathematics, Saint Xavier University, Chicago, Illinois, USA

**Keywords:** Nonlinear physics, Periodic cross-kink wave solution, Lump wave solution, (2+1)-dimensional nonlinear Calogero–Bogoyavlenskii–Schiff equation

## Abstract

A bilinear form of the (2+1)-dimensional nonlinear Calogero–Bogoyavlenskii–Schiff (CBS) model is derived using a transformation of dependent variable, which contain a controlling parameter. This parameter can control the direction, wave height and angle of the traveling wave. Based on the Hirota bilinear form and ansatz functions, we build many types of novel structures and manifold periodic-soliton solutions to the CBS model. In particular, we obtain entirely exciting periodic-soliton, cross-kinky-lump wave, double kinky-lump wave, periodic cross-kinky-lump wave, periodic two-solitary wave solutions as well as breather style of two-solitary wave solutions. We present their propagation features via changing the existence parametric values in graphically. In addition, we estimate a condition that the waves are propagated obliquely for η≠0 , and orthogonally for η=0.

## 1 Introduction

The nonlinear partial differential equations (NPDEs) have remained a subject of international research interest in physics, chemistry, biology and nonlinear sciences, especially, in nonlinear optics, photonics, Bose-Einstein condensate, harbor and coastal designs (Bruzon et al., 2003; Peng, 2006; Kobayashi and Toda, 2006; Li and Chen, 2004; Wang and Yang, 2012; Chen and Ma, 2018; Wazwaz, 2008; Ullah et al., 2020; Roshid and Ma, 2018; Hossen et al., 2018; Ming et al., 2013; Roshid and Roshid, 2018; Khatun et. al., 2020). To realize the physical mechanism of phenomena for the NPDEs in physics and engineering, their exact solutions are highly investigated. One of a significant nonlinear evolution equation is the Calogero–Bogoyavlenskii–Schiff (CBS) equation, which extensively used in various purposes. The CBS model is developed via dissimilar techniques (Peng, 2006; Kobayashi and Toda, 2006; Bruzon et al., 2003) and obtained its exact solutions (Li and Chen, 2004; Wang and Yang, 2012; Chen and Ma, 2018; Wazwaz, 2008) via the dint of symbolic computation.

Let us consider the CBS model (Peng, 2006; Kobayashi and Toda, 2006; Bruzon et al., 2003) is

(1) $$\sigma u_{xt}+\beta u_{x}u_{xy}+\delta u_{y}u_{xx}+u_{xxxy}=0\text{,}$$

where t∈ℜ is the time and x,y∈ℜ are the spatial variables.

Recently many authors ware worked on the CBS Eq. (1). The multiple-soliton solutions of the CBS model were obtained by Wazwaz, (2008). Zhang et al. (Zhang et al., 2009) did research on the CBS equation and they established substantially abundant symmetries and symmetry reduction of the (2+1)-dimensional generalized CBS equation. Moreover, Wazwaz, (2010) formed multiple soliton solutions and multiple singular soliton solutions for the (2+1)-dimensional as well as the (3+1)-dimensional CBS equations. Quasi-periodic wave solutions for the (2+1)-dimensional generalized CBS equation was incorporated in literature by Wang and Yang (Wang and Yang (2012)). More recently, Chen and Ma (2018) explored lump wave solutions of the generalized CBS equation.

In this article, we aim to determine a new bilinear form and determine innovative periodic-soliton solutions, periodic cross-kink wave, cross-double kink-periodic wave, periodic two-solitary wave as well as breather style of two-solitary wave of the CBS model.

## 2 Bilinear forms of the Calogero-Bogoyavlenskii-Schiff equation

In this section, we shall build a bilinear form of the CBS Eq. (1). To do that, at first makes over the (2+1)-dimensional nonlinear CBS (1) into the bilinear forms through the dependent variable transformations (Wang 2012):

(2) $$u=\eta y+\frac{3}{\delta }{\left[\ln\;\tau \left(x,y,t\right)\right]}_{x}$$

The beyond (2+1)-dimensional nonlinear evolution (1) is drawn into the Hirota D-operator equivalence (Wang 2012) as:

(3) $$\left(\sigma D_{x}D_{t}+D_{x}^{3}D_{y}-3\eta D_{x}^{2}+c\right)\tau .\tau =0$$

Now, we consider the relation between Hirota D-operator and its bilinear form via

(4) $$\prod_{i=1}^{N}D_{x_{i}}^{n_{i}}\tau .\upsilon ={\prod_{i=1}^{N}\left(\frac{\partial }{\partial x_{i}}-\frac{\partial }{\partial {\bar{x}}_{i}}\right)}^{n_{i}}\tau \left(x\right)\upsilon \left(\bar{x}\right){|}_{\bar{x}=x}\text{,}$$

where $x=\left(x_{1},\;\;\cdots \;\;\;\cdots ,\;\;x_{N}\right),\;\;\bar{x}=\left({\bar{x}}_{1},\;\;\cdots \;\;\;\cdots ,\;\;{\bar{x}}_{N}\right)$ nonzero vectors and $\;n_{1},\;\cdots \;,\;n_{N}$ are arbitrary nonnegative integers. Under formula (4), (3) can be converted (Wang 2012) to

(5) $$\tau \left(2\sigma \tau_{xt}+2\tau_{xxx}-{3\eta \tau_{xx}}^{2}\right)-2\sigma \tau_{x}\tau_{t}+6\tau_{xx}\tau_{xy}-6\tau_{x}\tau_{xxy}-2\tau_{xx}\tau_{y}+6\tau_{x}^{2}+c\tau^{2}=0$$

## 3 Solutions of the Calogero-Bogoyavlenskii-Schiff (CBS) equations

In this section, we present the dynamical behaviors of soliton solutions such as lump wave, multi-lump wave, interaction between kink and lump waves, and interactions of multi-lump and periodic wave for the CBS model in various subsections.

### 3.1 Lump solutions of the CBS equations

Through the support of the symbolic computational software Maple, we are going to determine positive quadratic solution to the CBS equation from its bilinear arrangement. Upon the 2-dimensional universe, a result elaborate summing of one square does not produce exact lump wave, which are reasonably local in every direction in the universe, under the relation (2). Consequently, we consider the trial solution of the sum of square of two linear polynomials as follows (Chen and Ma, 2018; Roshid and Ma, 2018):

(6) $$\tau =g^{2}+h^{2}+\ell_{9},\;\text{where}\;g\left(x,y,t\right)=\ell_{1}x+\ell_{2}y+\ell_{3}t+\ell_{4},\;h\left(x,y,t\right)=\ell_{5}x+\ell_{6}y+\ell_{7}t+\ell_{8}\text{.}$$

where $\ell_{i},\;1\leq i\leq 9,$ are real physical constraints to be obtained.

Putting (6) and (2) into Eq. (5), and solving for unfamiliar constraints $\ell_{i};\left(i=1,2,\ldots \ldots ,9\right)$ produces two set of constraint equations:

$$\left\{\ell_{1}=\ell_{1},\ell_{2}=-\frac{\ell_{6}\ell_{5}}{\ell_{1}},a_{3}=\frac{3\eta \ell_{1}}{\sigma },\ell_{4}=\ell_{4},\ell_{5}=\ell_{5},\ell_{6}=\ell_{6},\ell_{7}=\frac{3\eta \ell_{5}}{\sigma },\ell_{8}=\ell_{8},\ell_{9}=\ell_{9}\right\}\;\text{and}\;\left\{\ell_{1}=I\ell_{5},\ell_{2}=\ell_{2},\ell_{3}=\frac{3\eta I\ell_{5}}{\sigma },\ell_{4}=\ell_{4},\ell_{5}=\ell_{5},\ell_{6}=\ell_{6},\ell_{7}=\frac{3\eta \ell_{5}}{\sigma },\ell_{8}=\ell_{8},\ell_{9}=\ell_{9}\right\}\text{.}$$

Thus the solutions are

(7) $$u\left(x,t\right)=\eta y+\frac{3}{\delta }{\left(\ln\;\tau \right)}_{x}\text{,}$$

where $\tau ={\left(\ell_{1}x-\frac{\ell_{6}\ell_{5}}{\ell_{1}}y+\frac{3\eta \ell_{1}}{\sigma }t+\ell_{4}\right)}^{2}+{\left(\ell_{5}x+\ell_{6}y+\frac{3\eta \ell_{5}}{\sigma }+\ell_{8}\right)}^{2}+\ell_{9}$, $\ell_{1},\sigma \neq 0$ and $\ell_{1},\ell_{4},\ell_{5},\ell_{6},\ell_{8},\ell_{9}$ are random constants. The result (7) is to link with six arbitrary parameters $\ell_{1},\ell_{4},\ell_{5},\ell_{6},\ell_{8},\ell_{9}$, in turn to a kind of lump solutions of the CBS equations under the condition $\ell_{1}\neq 0,\ell_{9}>0$.

And

(8) $$u\left(x,t\right)=\eta y+\frac{3}{\delta }{\left(\ln\;\tau \right)}_{x}$$

where $\tau ={\left(I\ell_{5}x+\ell_{2}y+\frac{3\eta I\ell_{5}}{\sigma }t+\ell_{4}\right)}^{2}+{\left(\ell_{5}x+\ell_{6}y+\frac{3\eta \ell_{5}}{\sigma }t+\ell_{8}\right)}^{2}+\ell_{9}\text{,}$σ≠0 and $\ell_{2},\ell_{4},\ell_{5},\ell_{6},\ell_{8},\ell_{9}$ are random constants. This is a class of complex solutions, turned into lump solutions with the conditions $\ell_{2}\ell_{5}\neq 0,\ell_{9}>0\text{,}$.

**Figure 1:**
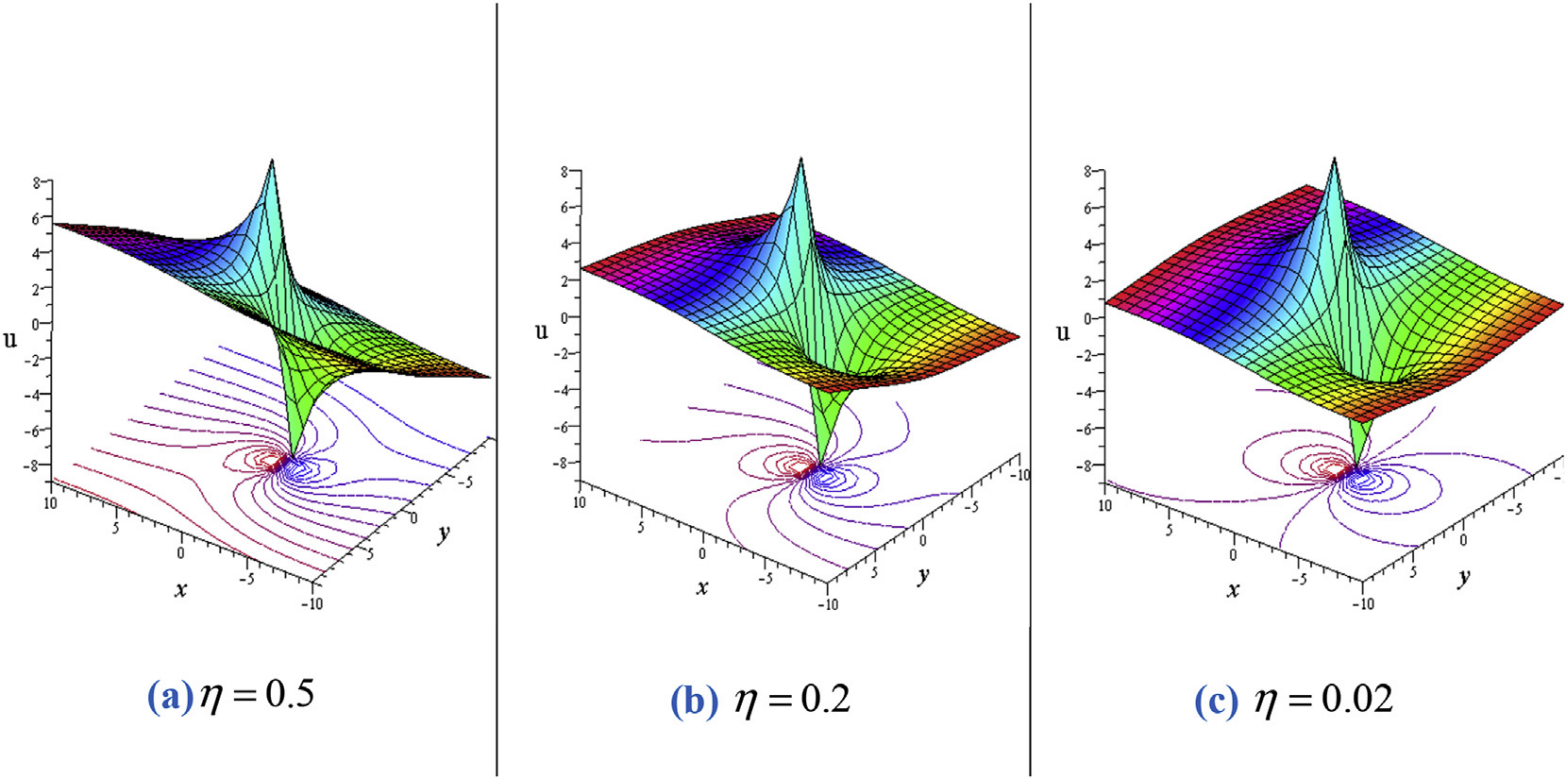
Profile of the Eq. (7) for $\ell_{1}=\ell_{4}=\ell_{6}=\ell_{8}=\ell_{9}=1,\sigma =1,\delta =0.3$: 3D plot (upper) and corresponding contour plot (below) at t=0 where images (a) for η=0.5, (b) for η=0.2 and (c) for η=0.02.

For solution Eq. (7), when η≠0then the lump wave solution gives one lump with an angle α$\left(\alpha \neq 90^{o}\right)$ to the water surface and has a deep hole at (−1.707723539,−0.02953994460)and a highest peak (−0.2922764605,0.02953994460). In this case, there are other two critical points at (2.426619521,3.352867629) and (−4.426619521,−3.352867629) through which flow of fluid particles are zero. It is evidently clear that the above four critical points has no any flow (see the contour plots of Figure 1(a) and (b)). Angle between the water surface and lump come to perpendicular as η→0 and ultimately orthogonal for η=0. When η=0, then the lump wave solution gives only one lump perpendicular with the water surface, and reduces to two critical points (instant of four) for a deep hole at (−1.707106781,0) and a highest peak at (−0.2928932188,0).

**Figure 2:**
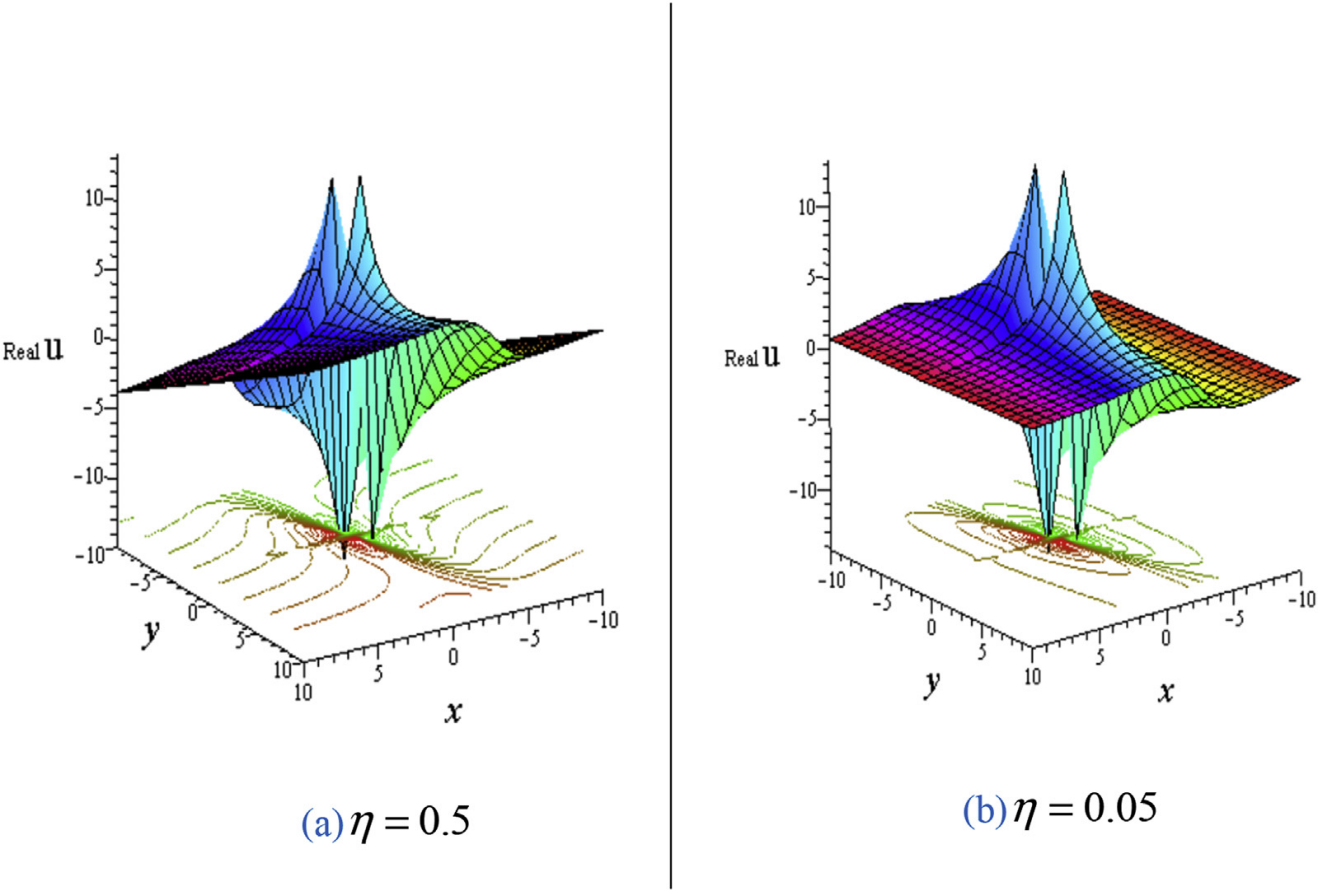
Profile of the Eq. (8) for$\ell_{4}+\ell_{5}+\ell_{6}+\ell_{8}=\ell_{9}=1,\sigma =1,\delta =0.3$: 3D plot (upper) and corresponding contour plot (below) at t=0 where images (a) Real part of the Eq. (8) and (b) Imaginary part of the Eq. (8).

For solution Eq. (8), each of real and imaginary part gives the couple lump solutions (See Figure 2) whose characteristics are similar to the single lump. Also, angle between the lump and water surface can be controlled in the similar to Fig. (1).

### 3.2 Interaction between kink and lump waves solutions of CBS equation

We now pick the trial solution for the superposition of two quadratic polynomials and an exponential function:

(9) $$\tau =g^{2}+h^{2}+\ell_{9}+h_{1}e^{k}$$

where$g\left(x,y,t\right)=\ell_{1}x+\ell_{2}y+\ell_{3}t+\ell_{4}$.$h\left(x,y,t\right)=\ell_{5}x+\ell_{6}y+\ell_{7}t+\ell_{8,}$

$k\left(x,y,t\right)=\ell_{10}x+\ell_{11}y+\ell_{12}t$ and $\ell_{i}$, 1≤i≤12 which are real parameters to be determinant.

Substituting Eq. (9) into Eq. (5) and solving for unknown parameters $\ell_{i};\left(i+1,2,\ldots \ldots ,12\right)$yields one set of constraint:

$$\left\{\ell_{1}=0,\ell_{2}=-\frac{\sigma \ell_{3}}{\ell_{10}^{2}},\ell_{3}=\ell_{3},\ell_{4}=\ell_{4},\ell_{5}=0,\ell_{6}=-\frac{\sigma \ell_{7}}{\ell_{10}^{2}},\ell_{7}=\ell_{7},\ell_{8}=\ell_{8},\ell_{9}=\ell_{9},\ell_{10}=\ell_{10},\ell_{11}=-\frac{\sigma \ell_{12}}{\ell_{10}^{2}},\ell_{12}=\ell_{12},h_{1}=h_{1}\right\}$$

Thus the solution

(10) $$u\left(x,t\right)+\eta y+\frac{3}{\delta }{\left(\ln\;\tau \right)}_{x}\text{,}$$

where $\tau +{\left(-\frac{\sigma \ell_{3}}{\ell_{10}^{2}}y+\ell_{3}t+\ell_{4}\right)}^{2}+{\left(-\frac{\sigma \ell_{7}}{\ell_{10}^{2}}y+\ell_{7}t+\ell_{8}\right)}^{2}+\ell_{9}+h_{1}e^{\left(\ell_{10}x-\frac{\sigma \ell_{12}}{\ell_{10}^{2}}y+\ell_{12}t\right)}$;$\ell_{10}\neq 0$ and

**Figure 3:**
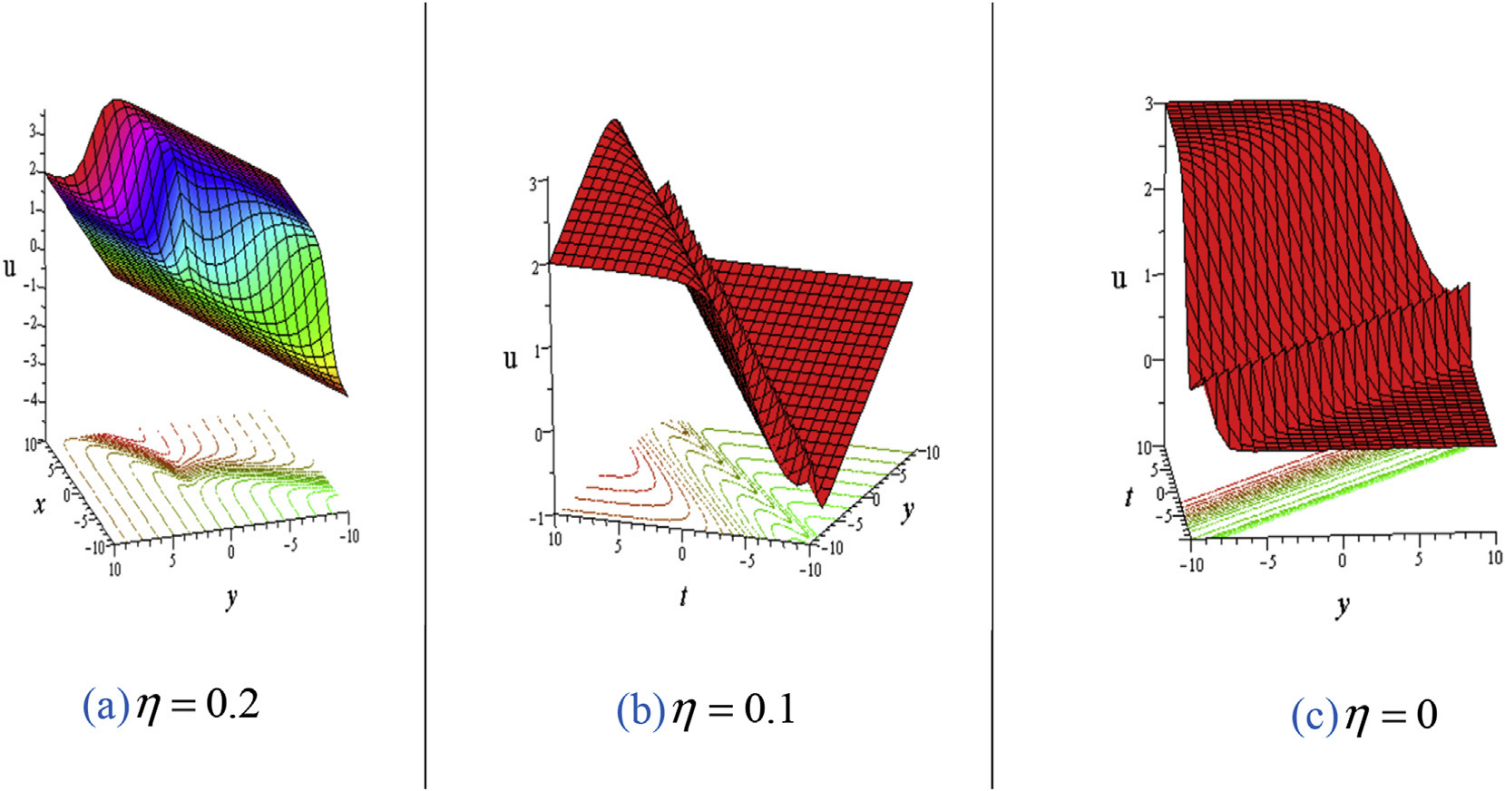
Profile of the Eq. (10) for $\ell_{1}=\ell_{5}=0,\ell_{3}=\ell_{4}=\ell_{7}=\ell_{8}=\ell_{9}=\ell_{12}=h_{1}=\sigma =1,\delta =:2$: 3D plot (upper) and corresponding contour plot (below) at t=0 where images (a) for η=0.2, (b) for η=0.1 and (c) for η=0.

$\ell_{3},\ell_{4},\ell_{7},\ell_{8},\ell_{9},\ell_{10},\ell_{12}$are arbitrary constants. We see that angle of flow can be controlled via the parameter η, which explained in the previous subsection 3.1. The motion of particle describes in a curvy path for η≠0, but tend to diminish into a linear path as η→0 and exactly through line for η+0(see contour plot of Figure 3).

### 3.3 Multi lump solutions of the CBS equation

Let us pick the trial solution for the superposition of two exponential functions and a cosine function:

(11) $$\tau +e^{-d_{1}g}+h_{1}e^{d_{1}g}+h_{2}\left(\cos\left(d_{2}h\right)\right)\;\text{where}\;g\left(x,y,t\right)+x+\ell_{1}y+w_{1}t,\;h\left(x,y,t\right)+x+\ell_{2}y+w_{2}t\text{,}$$

where $\ell_{1},\ell_{2},w_{1},w_{2}$are real parameters to be calculated.

Inserting Eq. (11) into Eq. (5), and solving for the unknown parameters $\ell_{1},\;\ell_{2},\;w_{1},\;w_{2},\;d_{1},\;d_{2}$ yield a set of constraints:

$$\left\{\ell_{1}=-\frac{1}{2}\frac{\sigma w_{2}-3\eta }{d_{1}^{2}},\ell_{2}=0,d_{1}=d_{1},d_{2}+d_{2},h_{1}+-\frac{1}{2}\frac{h_{2}^{2}d_{2}^{2}}{3d_{1}^{2}+d_{2}^{2}},h_{2}+h_{2},\;w_{1}+\frac{1}{2}\frac{3\eta d_{1}^{2}+\sigma w_{2}d_{1}^{2}+3\eta d_{1}^{2}-\sigma d_{2}^{2}w_{2}}{d_{1}^{2}\sigma },w_{2}=w_{2}\right\}$$

**Figure 4:**
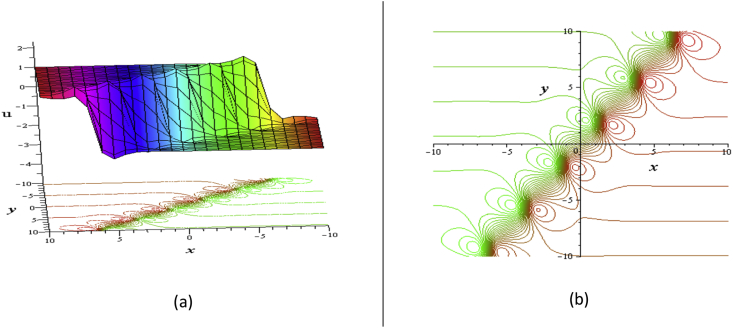
Profile of the Eq. (12) for $\ell_{1}=d_{1}=d_{2}=h_{1}=h_{2}=1,\sigma =2,\delta =2,\eta =0.05:$ (a) 3D plot (upper) and contour plot (below) and (b) contour plot at t = 0.

Thus the solution

(12) $$u\left(x,t\right)=\eta y+\frac{3}{\delta }{\left(\ln\;\tau \right)}_{x}\text{,}$$

where $\tau =e^{-d_{1}\left(x-\frac{1}{2}\frac{\sigma w_{2}-3\eta }{d_{1}^{2}}y+w_{1}t\right)}-\frac{1}{2}\frac{h_{2}^{2}d_{2}^{2}}{3d_{1}^{2}+d_{2}^{2}}e^{d_{1}\left(x-\frac{\sigma w_{2}-3\eta }{d_{1}^{2}}y+w_{1}t\right)}+h_{2}\left(\cos\left(d_{2}\left(x+w_{2}t\right)\right)\right)$, $d_{1},\sigma \neq 0$ and $w_{1}=\frac{1}{2}\frac{3\eta d_{1}^{2}+\sigma w_{2}d_{1}^{2}+3\eta d_{1}^{2}-\sigma d_{2}^{2}w_{2}}{d_{1}^{2}\sigma }$,$w_{2},d_{1},d_{2},\sigma$are arbitrary constants. The direction of the angle of the flow can be controlled depending on the values of η, which discussed in the previous subsection 3.1. The solution (12) has the real shape as in the Figure 4.

### 3.4 Interaction of the multi-lump and periodic solutions of the CBS equation

Let us take the trial solution for the superposition of the sum and product of sine, cosine and their hyperbolic functions:

(13) $$\tau =1+\cosh\left(\zeta_{1}\right)\sigma_{1}\;\cos\left(\zeta_{2}\right)+\cosh\left(\zeta_{1}\right)\sigma_{2}\;\sin\left(\zeta_{2}\right)+\sigma_{3}\;\sinh\left(\zeta_{1}\right)+\sigma_{4}\;\cosh\left(\zeta_{2}\right)$$

where $\zeta_{1}\left(x,y,t\right)=\ell_{1}x+{℘}_{1}y+w_{1}t,$$\zeta_{2}\left(x,y,t\right)=\ell_{2}x+{℘}_{2}y+w_{2}t$ and $\ell_{1},\;\ell_{2},\;{℘}_{1},\;{℘}_{2},\;w_{1},w_{2}$, are parameters to be calculated.

Putting Eq. (13) into Eq. (5), and resolving for unknown parameters $\ell_{1},\;\ell_{2},\;{℘}_{1},\;{℘}_{2},\;w_{1},\;w_{2}$ yield eight set of constraints:

**Set-1:**

$$\left\{\ell_{1}=\ell_{1},\ell_{2}=\ell_{2},{℘}_{1}=-\frac{1}{4}\frac{3\eta \ell_{1}+\sigma w_{1}}{\ell_{1}^{2}},{℘}_{2}=\frac{1}{8}\frac{-9\eta \ell_{1}^{3}+3\sigma w_{1}\ell_{1}^{2}-3\eta \ell_{1}\ell_{2}^{2}+qw_{1}\ell_{2}^{2}}{\ell_{1}^{3}\ell_{2}},\sigma_{1}=I\sigma_{2},\sigma_{2}=\sigma_{2},\sigma_{3}=0,\sigma_{4}=0,w_{1}=w_{1},w_{2}=-\frac{1}{8}\frac{-6\ell_{2}^{2}\sigma w_{1}\ell_{1}^{2}+3\eta \ell_{1}\ell_{2}^{4}-\sigma w_{1}\ell_{2}^{4}-6\ell_{2}^{2}\eta \ell_{1}^{3}-9\ell_{1}^{5}\eta +3\ell_{1}^{4}\sigma w_{1}}{\ell_{1}^{3}\ell_{2}\sigma }\right\}$$

**Set-2:**

$$\left\{\ell_{1}=0,\ell_{2}=\ell_{2},{℘}_{1}=-\frac{\sigma w_{1}}{\ell_{2}^{2}},{℘}_{2}=0,\sigma_{1}=0,\sigma_{2}=0,\sigma_{3}=\sigma_{3},\sigma_{4}=\sigma_{4},w_{1}=w_{1},w_{2}=\frac{3\eta \ell_{2}}{\sigma }\right\},$$

**Set-3:**

$$\left\{\ell_{1}=0,\ell_{2}=\ell_{2},{℘}_{1}=\frac{\sigma w_{1}}{\ell_{2}^{2}},{℘}_{2}=\frac{-3\eta \ell_{2}+\sigma w_{2}}{\ell_{2}^{2}},\sigma_{1}=I\sigma_{2},\sigma_{2}=\sigma_{2},\sigma_{3}=\sigma_{3},\sigma_{4}=0,w_{1}=w_{1},w_{2}=w_{2}\right\},$$

**Set-4:**

$$\left\{\ell_{1}=\ell_{1},\ell_{2}=-\ell_{1},{℘}_{1}=-\frac{-3\eta \ell_{1}+\sigma w_{1}}{\ell_{1}^{2}},{℘}_{2}=-\frac{-3\eta \ell_{1}+\sigma w_{1}}{\ell_{1}^{2}},\sigma_{1}=0,\sigma_{2}=0,\sigma_{3}=I\sigma_{4},\sigma_{4}=\sigma_{4},w_{1}=w_{1},w_{2}=\frac{-6\eta \ell_{1}+\sigma w_{1}}{\sigma }\right\},$$

**Set-5:**

$$\left\{\ell_{1}=\ell_{1},\ell_{2}=\ell_{1},{℘}_{1}=-\frac{-3\eta \ell_{1}+\sigma w_{1}}{\ell_{1}^{2}},{℘}_{2}=\frac{-3\eta \ell_{1}+\sigma w_{1}}{\ell_{1}^{2}},\sigma_{1}=0,\sigma_{2}=0,\sigma_{3}=I\sigma_{4},\sigma_{4}=\sigma_{4},w_{1}=w_{1},w_{2}=-\frac{-6\eta \ell_{1}+\sigma w_{1}}{\sigma }\right\},$$

**Set-6:**

$$\left\{\ell_{1}=\ell_{1},\ell_{2}=0,{℘}_{1}=0,{℘}_{2}={℘}_{2},\sigma_{1}=\sigma_{1},\sigma_{2}=\sigma_{2},\sigma_{3}=0,\sigma_{4}=\sigma_{4},w_{1}=\frac{3\eta \ell_{1}}{\sigma },w_{2}=-\frac{\ell_{1}^{2}{℘}_{2}}{\sigma }\right\},$$

**Set-7:**

$$\left\{\ell_{1}=I\ell_{2},\ell_{2}=\ell_{2},{℘}_{1}=-\frac{1}{4}\frac{3\eta I\ell_{2}-\sigma w_{1}}{\ell_{2}^{2}},{℘}_{2}={℘}_{2},\sigma_{1}=I\sigma_{2},\sigma_{2}=\sigma_{2},\sigma_{3}=0,\sigma_{4}=0,w_{1}=w_{1},w_{2}=\frac{\ell_{2}\left(3\eta +4{℘}_{2}\ell_{2}\right)}{\sigma }\right\},$$

**Set-8:**

$$\left\{\ell_{1}=\ell_{1},\;\ell_{2}=\ell_{2},\;{℘}_{1}=0,\;{℘}_{2}=0,\;\sigma_{1}=\sigma_{1},\;\sigma_{2}=\sigma_{2},\;\sigma_{3}=\sigma_{3},\;\sigma_{4}=\sigma_{4},\;w_{1}=\frac{3\eta \ell_{1}}{\sigma },\;w_{2}=\frac{3\eta \ell_{2}}{\sigma }\right\}$$

For the **Set-1**, the solution

(14) $$u\left(x,t\right)=\eta y+\frac{3}{\delta }{\left(\ln\;\tau \right)}_{x}\text{,}$$

where

$$\tau =1+\cosh\left(\ell_{1}x-\frac{1}{4}\frac{-3\eta \ell_{1}+\sigma w_{1}}{\ell_{1}^{2}}y+w_{1}t\right)I\sigma_{2}\;\cos\left(\ell_{2}x+{℘}_{2}y+w_{2}t\right)+\cosh\left(\ell_{1}x-\frac{1}{4}\frac{-3\eta \ell_{1}+\sigma w_{1}}{\ell_{1}^{2}}y+w_{1}t\right)\sigma_{2}\;\sin\left(\ell_{2}x+{℘}_{2}y+w_{2}t\right),$$

with$w_{2}=-\frac{1}{8}\frac{-6\ell_{2}^{2}\sigma w_{1}\ell_{1}^{2}+3\eta \ell_{1}\ell_{2}^{4}-\sigma w_{1}\ell_{2}^{4}-6\ell_{2}^{2}\eta \ell_{1}^{3}-9\ell_{1}^{5}\eta +3\ell_{1}^{4}\sigma w_{1}}{\ell_{1}^{3}\ell_{2}\sigma },$${℘}_{2}=\frac{1}{8}\frac{-9\eta \ell_{1}^{3}+3\sigma w_{1}\ell_{1}^{2}-3\eta \ell_{1}\ell_{2}^{2}+qw_{1}\ell_{2}^{2}}{\ell_{1}^{3}\ell_{2}}$, σ≠0, $\ell_{1},\ell_{2},\;w_{1}$and $\sigma_{2}$ are arbitrary constants.

**Figure 5:**
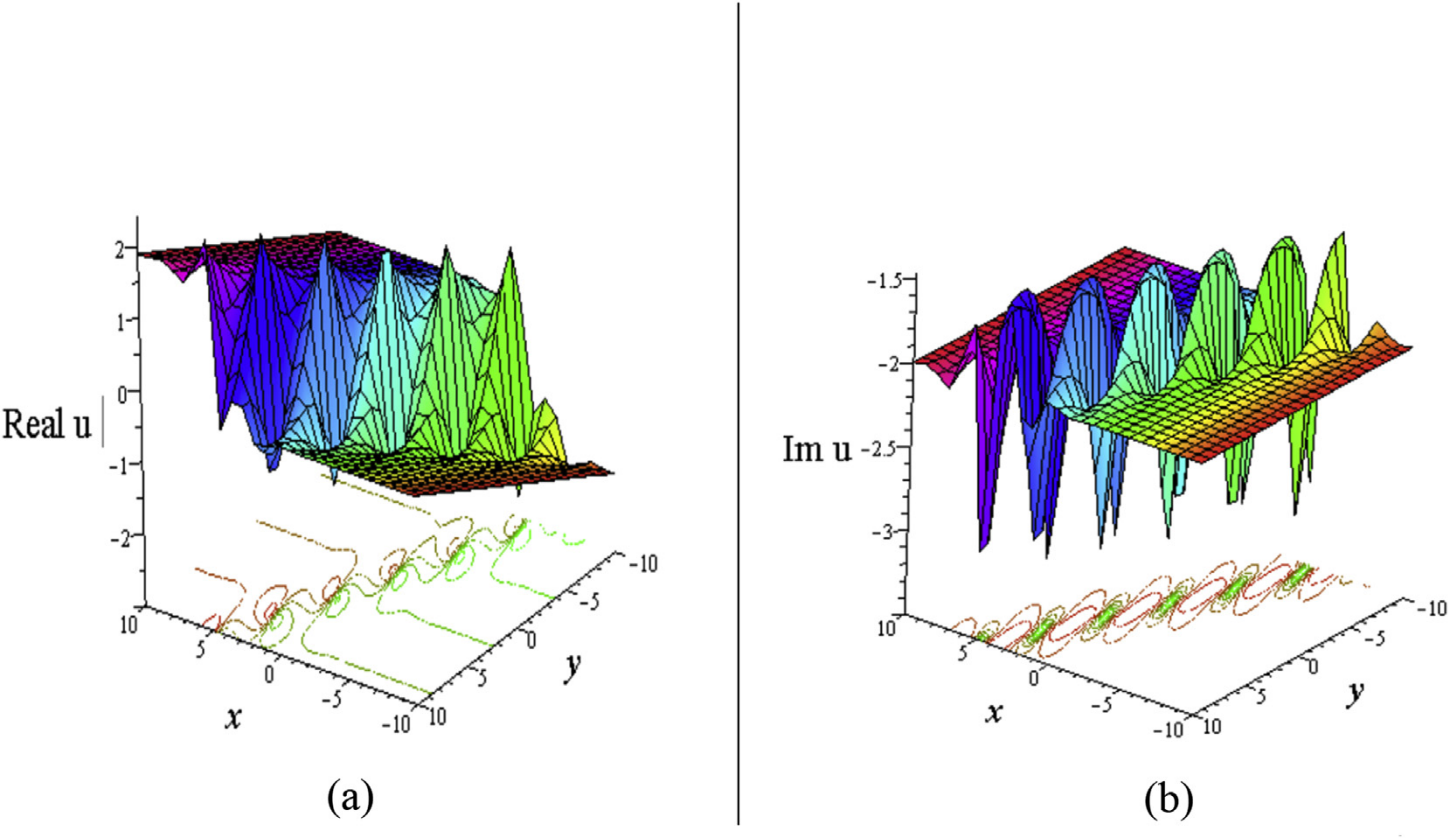
Profile of the Eq. (14) for $\sigma =-1,\eta =0.09,w_{1}=-2,a_{1}=1,a_{2}=\sigma_{2}=2,\sigma_{3}=\sigma_{4}=0$,δ=3: 3D plots (upper) and corresponding contour plots (below) at t=0 where images (a) Real part of the Eq. (14) and (b) Imaginary part of the Eq. (14).

The solution Eq. (14) from the combinations of hyperbolic and sinusoidal functions gives kinky wave whose lumps occurs periodically, known as kinky-periodic-lump wave (Figure 5(a) for the real part, and Figure 5(b) for the imaginary part of the solution).

**Figure 6:**
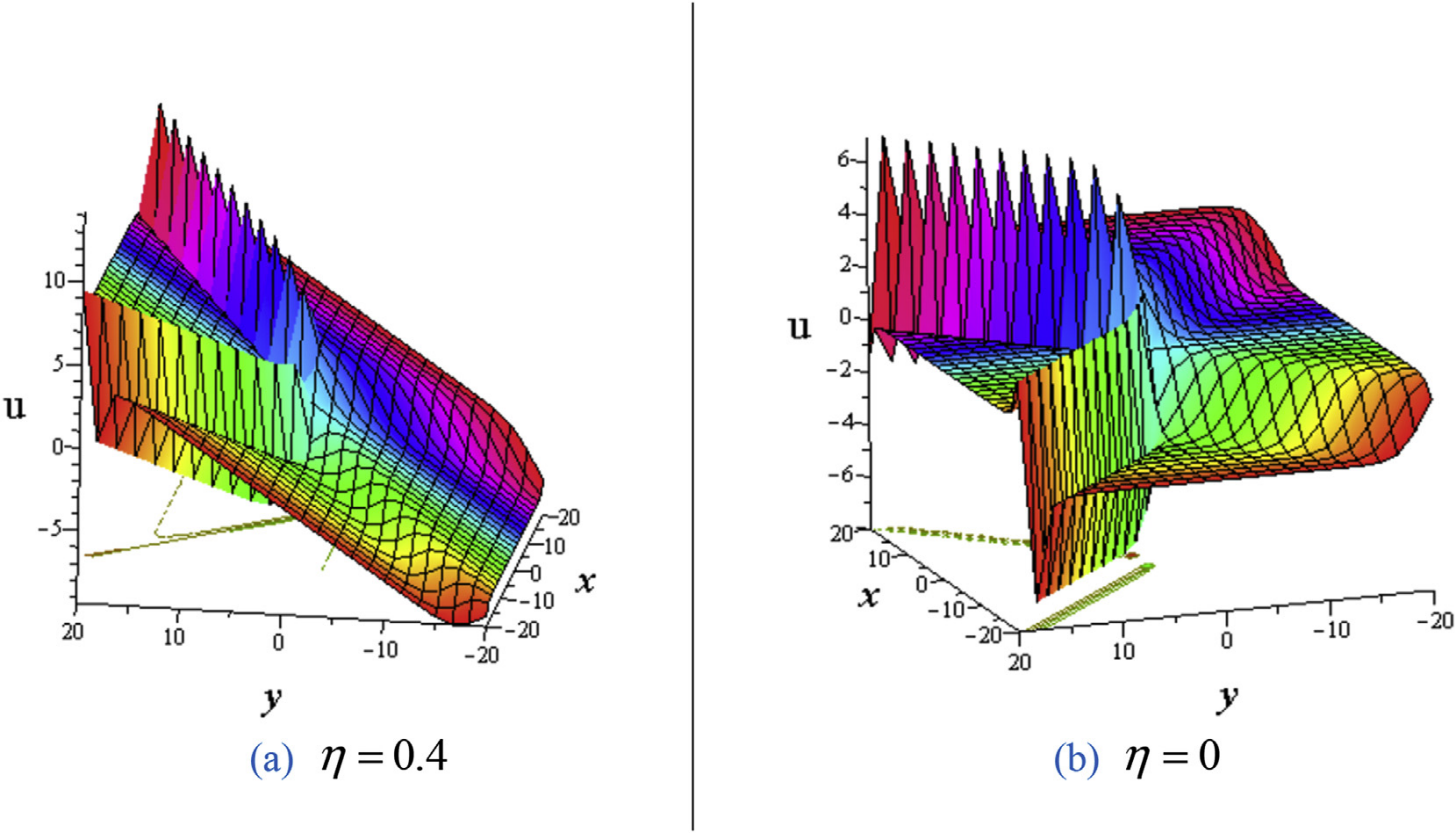
Profile of the Eq. (15) for $\ell_{i}={℘}_{2}=\sigma_{1}=\sigma_{2}=0,\;\ell_{2}=w_{1}=\sigma =\delta =\sigma_{4}=1,\;\eta =0.4:$ 3D plot (upper) and contour plot (below) at t=0 where images (a) for η=0.4and (b) for η=0.

For **the Set-2**, the solution

(15) $$u\left(x,t\right)=\eta y+\frac{3}{\delta }{\left(\ln\;\tau \right)}_{x},$$

where $\tau =1+\sigma_{3}\;\sinh\left(-\frac{\sigma w_{1}}{\ell_{2}^{2}}y+w_{1}t\right)+\sigma_{4}\;\cosh\left(\ell_{2}x+\frac{3\eta \ell_{2}}{\sigma }t\right)$,$\ell_{2},\sigma \neq 0$and $\ell_{2},w_{1},\sigma_{3},\sigma_{4}$ are arbitrary constants. For the solution Eq. (15), we obtain cross kinky-lump wave solution. The cross-kinky-lump wave propagate obliquely for η≠0(See Figure 6(a)), and orthogonally for η=0 with water surface (See Figure 6(b)).

For **the Set-3**, the solution

(16) $$u\left(x,t\right)=\eta y+\frac{3}{\delta }{\left(\ln\;\tau \right)}_{x},$$

where

$$\tau =1+\cosh\left(\frac{\sigma w_{1}}{\ell_{2}^{2}}y+w_{1}t\right)I\sigma_{2}\;\cos\left(\ell_{2}x+\frac{-3\eta \ell_{2}+\sigma w_{2}}{\ell_{2}^{2}}y+w_{2}t\right)+\cosh\left(\frac{\sigma w_{1}}{\ell_{2}^{2}}y+w_{1}t\right)\sigma_{2}\;\sin\left(\ell_{2}x+\frac{-3\eta \ell_{2}+\sigma w_{2}}{\ell_{2}^{2}}y+w_{2}t\right)+\sigma_{3}\;\sinh\left(\frac{\sigma w_{1}}{\ell_{2}^{2}}y+w_{1}t\right),\ell_{2}\neq 0$$

and $\ell_{2},\;\sigma_{2},\;\sigma_{3},\;w_{1}$are arbitrary constants.

**Figure 7:**
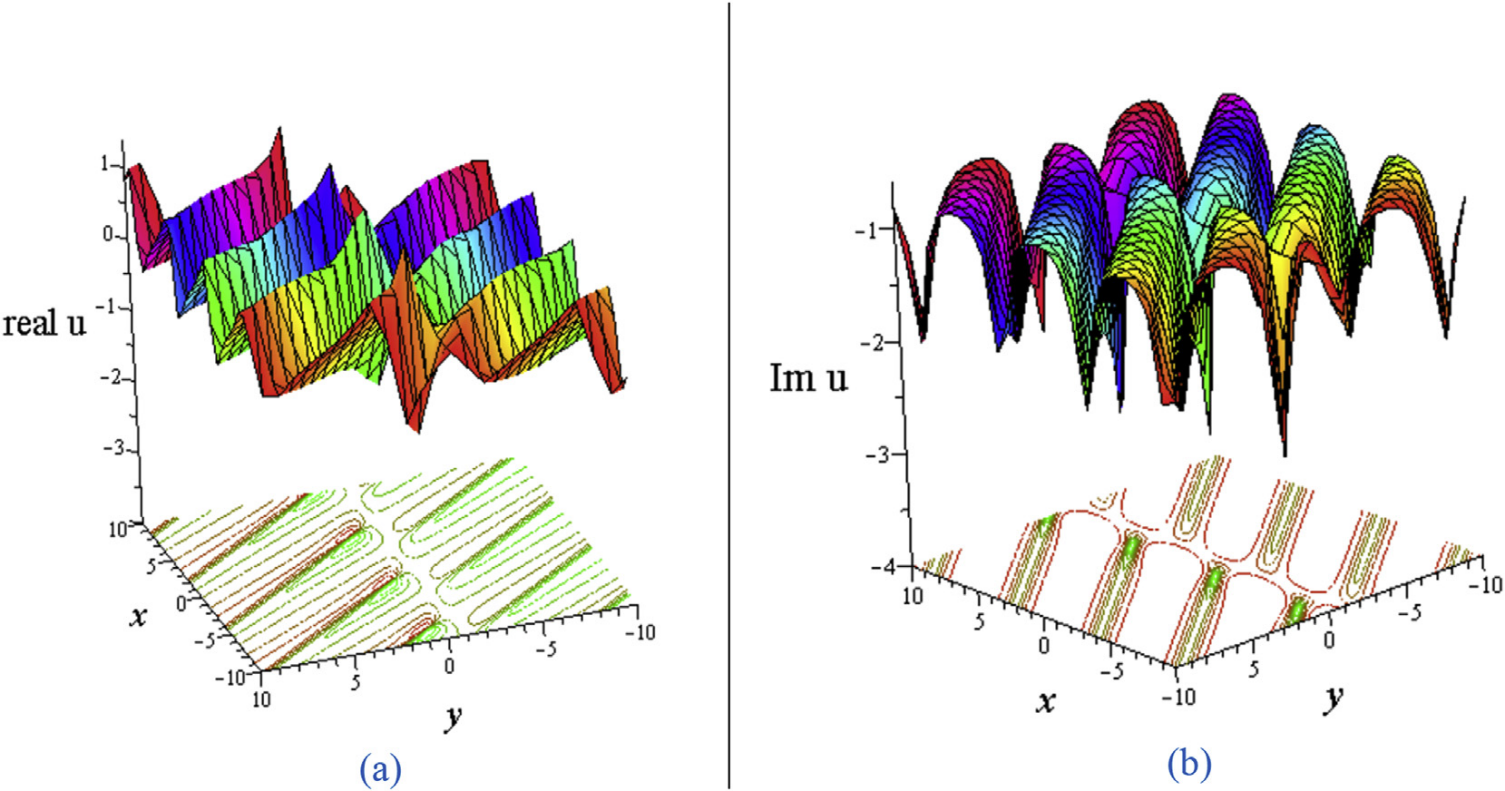
Profile of interaction of lump and periodic wave solution of the Eq. (16) for $\ell_{1}=\sigma_{4}=0,\;\ell_{2}=w_{1}=1$$w_{2}=\sigma =1,\sigma_{2}=2,\eta =0.04,\delta =3:$3D plot (upper) and corresponding contour plot (below) at t=0 where images (a) Real part of the Eq. (16) and (b) Imaginary part of the Eq. (16).

The solution Eq. (16) from the combinations of hyperbolic and sinusoidal functions gives the interaction of the periodic wave with a lump wave which known as periodic-lump wave (Figure 7(a) for the real part, and Figure 7(b) for the imaginary part).

For **the Set-4**, the solution

(17) $$u\left(x,t\right)=\eta y+\frac{3}{\delta }{\left(\ln\;\tau \right)}_{x},$$

where $\tau =1+I\sigma_{4}\;\sinh\left(a_{1}x-\frac{-3\eta \ell_{1}+\sigma w_{1}}{\ell_{1}^{2}}y+w_{1}t\right)+\sigma_{4}\;\cosh\left(-\ell_{1}x-\frac{-3\eta \ell_{1}+\sigma w_{1}}{\ell_{1}^{2}}y+\frac{-6\eta \ell_{1}+\sigma w_{1}}{\sigma }t\right)$,

$\ell_{1},\sigma \neq 0$ and $\ell_{1},w_{1}$are arbitrary constants.

**Figure 8:**
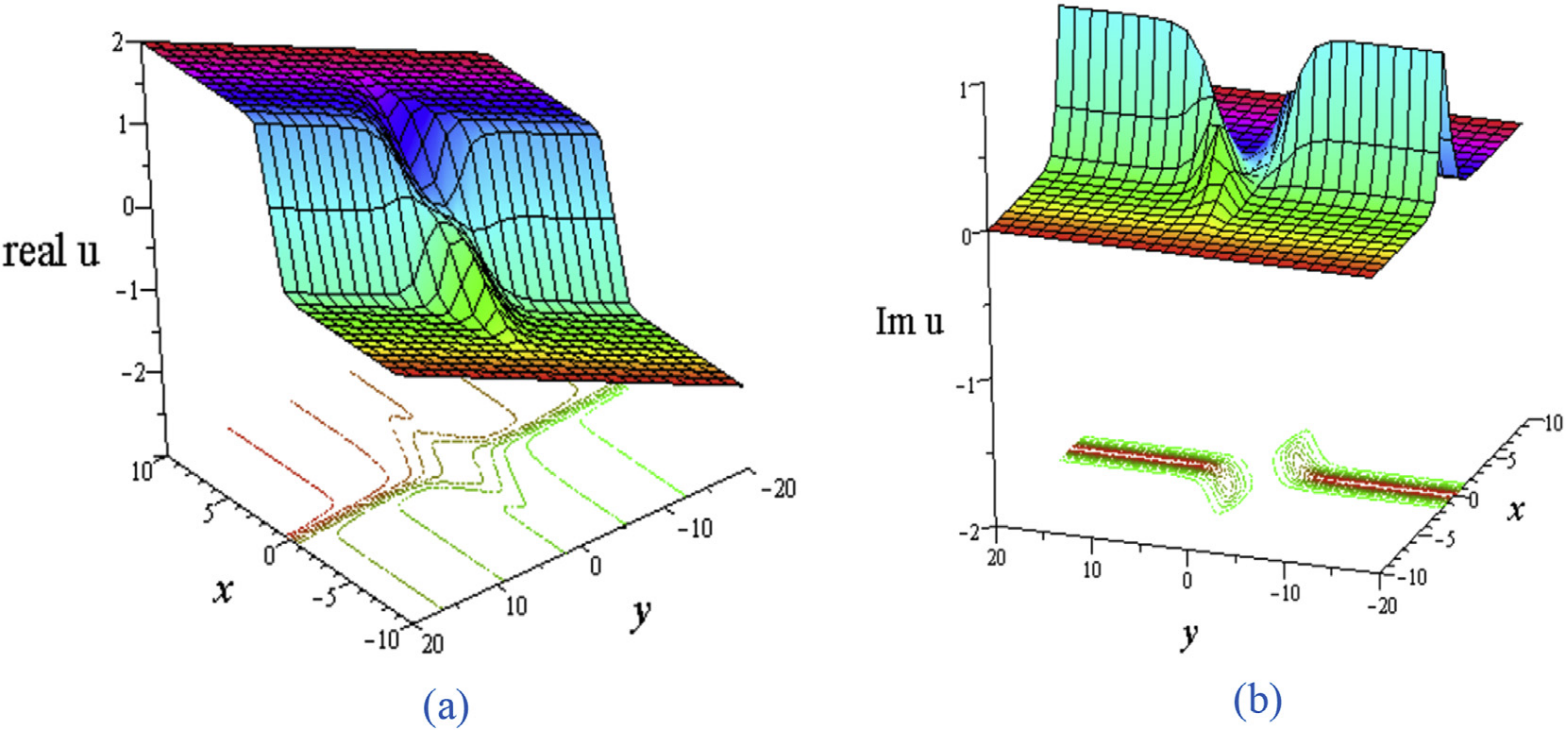
Profile of two bell solitons and two kink solitons of the Eq. (17) for $\ell_{1}=1,\ell_{2}=-1,w_{1}=\sigma =1,\sigma_{1}=\sigma_{2}=0,\sigma_{4}=0.05,\eta =0.05,$δ=3: 3D plot (upper) and corresponding contour plot (below) at t = 0 where images (a) Real part of Eq. (17) and (b) Imaginary part of Eq. (17).

The solution Eq. (17) comes from the hyperbolic functions only whose real part leads two bell wave separated at the origin and the imaginary part leads two kink wave separated at the origin (Figure 8(a) for the real part and Figure 8(b) for the imaginary part). The contour path of the motion of particles is drawn under the corresponding 3D plots.

For the **Set-5**, the solution

(18) $$u\left(x,t\right)=\eta y+\frac{3}{\delta }{\left(\ln\;\tau \right)}_{x},$$

where $\tau =1+I\sigma_{4}\;\sinh\left(\ell_{1}x-\frac{-3\eta \ell_{1}+\sigma w_{1}}{\ell_{1}^{2}}y+w_{1}t\right)+\sigma_{4}\;\cosh\left(\ell_{1}x+\frac{-3\eta \ell_{1}+\sigma w_{1}}{\ell_{1}^{2}}y-\frac{-6\eta \ell_{1}+\sigma w_{1}}{\sigma }t\right)$,

$\ell_{1},\sigma \neq 0$ and $\ell_{1},w_{1}$are arbitrary constants.

**Figure 9:**
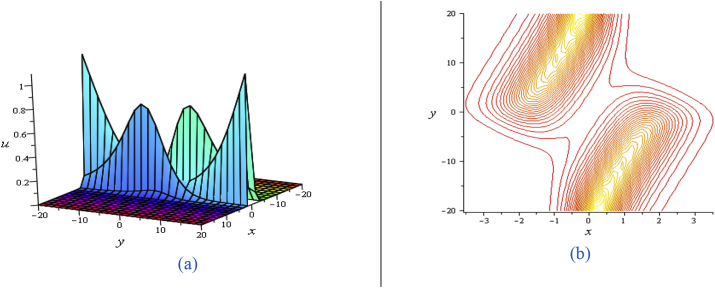
Profile of the Eq. (18) for $\ell_{1}=\ell_{2}=2,\sigma_{1}=\sigma_{2}=0,\sigma_{4}=0.05,w_{1}=\sigma =1,\eta =0.05,\delta =3:$(a) 3D plot (left), (b) contour plot (right) at t = 0.

The solution Eq. (18) comes only from hyperbolic functions whose real part gives two soliton separated at the origin (See Figure 9 and it corresponding contour plot) similar to the solution Eq. (17).

For **Set-6**, the solution

(19) $$u\left(x,t\right)=\eta y+\frac{3}{\delta }{\left(\ln\;\tau \right)}_{x},$$

where

$$\tau =1+\cosh\left(\ell_{1}x+\frac{3\eta \ell_{1}}{\sigma }t\right)\sigma_{1}\;\cos\left({℘}_{2}y-\frac{\ell_{1}^{2}{℘}_{2}}{\sigma }t\right)+\cosh\left(\ell_{1}x+\frac{3\eta \ell_{1}}{\sigma }t\right)\sigma_{2}\;\sin\left({℘}_{2}y-\frac{\ell_{1}^{2}{℘}_{2}}{\sigma }t\right)+\sigma_{4}\;\cos\left({℘}_{2}y-\frac{\ell_{1}^{2}{℘}_{2}}{\sigma }t\right)\text{,}$$

σ≠0 and $\ell_{1},{℘}_{2},\sigma_{1},\sigma_{2},\sigma_{4}$ are arbitrary constants.

**Figure 10:**
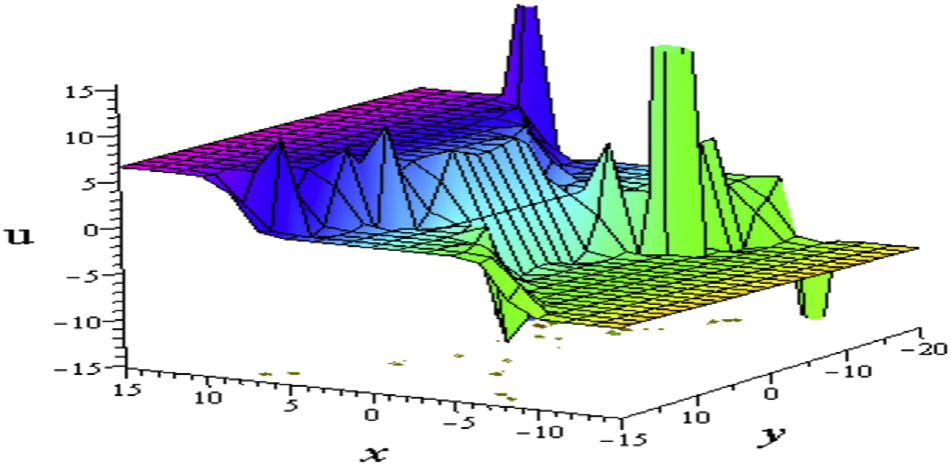
Profile of cross-double kinky-lump wave of the Eq. (19), for $\ell_{1}=\sigma_{1}=2,\ell_{2}={℘}_{1}=0,{℘}_{2}=,\sigma =1,\sigma_{2}=3,\sigma_{3}=0,\eta =0.03,\delta =1:$ 3D plot (upper) and corresponding contour plot (below) at t=0.

This solution Eq. (19) from the combinations of hyperbolic and sinusoidal functions gives interaction of double kinky wave, whose lump waves occurs periodically known as the cross-double kinky-lump wave (See Figure 10).

For **Set-7**, the solution

(20) $$u\left(x,t\right)=\eta y+\frac{3}{\delta }{\left(\ln\;\tau \right)}_{x},$$

where

$$\tau =1+\cosh(I\ell_{2}x-\frac{1}{4}\frac{3\eta I\ell_{2}-\sigma w_{1}}{\ell_{2}^{2}}y+w_{1}t)I\sigma_{2}\;\cos(\ell_{2}x+{℘}_{2}y+\frac{\ell_{2}(3\eta +4{℘}_{2}\ell_{2})}{\sigma }t+\cosh(I\ell_{2}x-\frac{1}{4}\frac{3\eta I\ell_{2}-\sigma w_{1}}{\ell_{2}^{2}}y+w_{1}t)\sigma_{2}\;\sin(\ell_{2}x+{℘}_{2}y+\frac{\ell_{2}(3\eta +4{℘}_{2}\ell_{2})}{\sigma }t),\ell_{2},\sigma \neq 0$$

and $\ell_{2},{℘}_{2},\sigma_{2},w_{1}$ are arbitrary constants.

**Figure 11:**
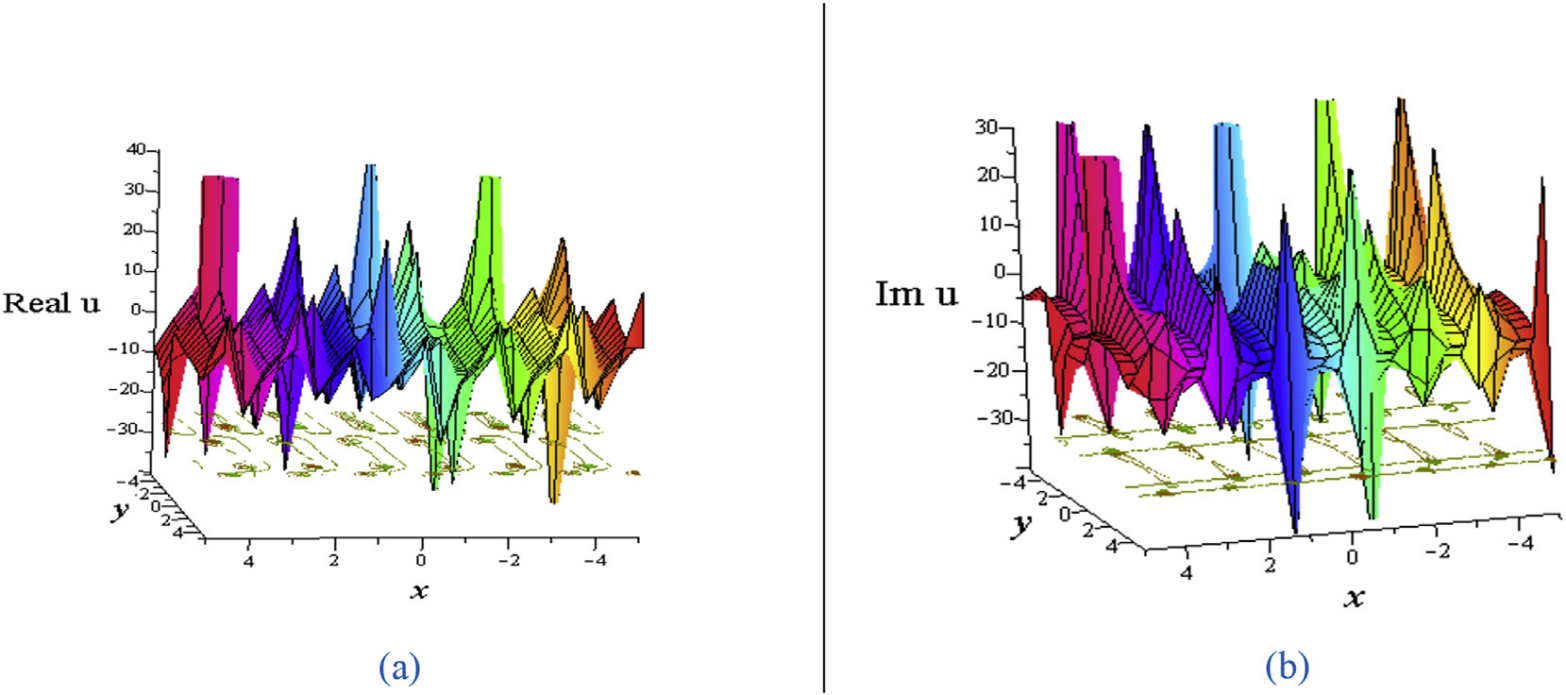
Profile of multi-periodic lump wave of the Eq. (20)$\ell_{2}=2,\;{℘}_{1}=1,\;\sigma_{2}=2,\;\sigma_{3}=\sigma_{4}=0,w_{1}=\sigma =1,\eta =0.3,\delta =1:$ 3D plot (upper) and corresponding contour plot (below) at t=0 where images (a) Real part of Eq. (20) and (b) Imaginary part of Eq. (20).

The solution Eq. (20) from the combinations of hyperbolic and sinusoidal functions gives interaction of periodic wave with a lump wave known as multi-periodic-lump wave (Figure 11(a, b)). The contour path of the motion of particles is traded under the corresponding 3D plots.

For **Set-8**, the solution

(21) $$u\left(x,t\right)=\eta y+\frac{3}{\delta }{\left(\ln\;\tau \right)}_{x},$$

**Figure 12:**
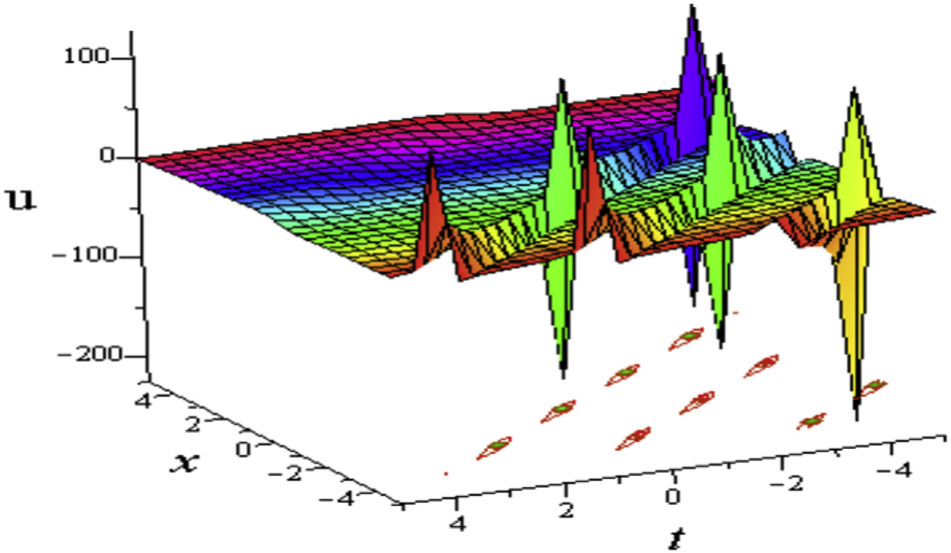
Profile of the Eq. (21) for $\ell_{1}=\ell_{2}=0,{℘}_{1}={℘}_{2}=0,\sigma_{1}=\sigma_{2}=\sigma_{3}=\sigma_{4}=1,\sigma =1,\eta =0.3,$δ=1:3D plot (upper) and contour plot (below) at t = 0.

$\tau =1+\cosh\left(\ell_{1}x+\frac{3\eta \ell_{1}}{\sigma }t\right)\sigma_{1}\;\cos\left(\ell_{2}x+\frac{3\eta \ell_{2}}{\sigma }t\right)+\cosh\left(\ell_{1}x+\frac{3\eta \ell_{1}}{\sigma }t\right)\sigma_{2}\;\sin\left(\ell_{2}x+\frac{3\eta \ell_{2}}{\sigma }t\right)+\sigma_{3}\;\sinh\left(\ell_{1}x+\frac{3\eta \ell_{1}}{\sigma }t\right)+\sigma_{4}\;\cosh\left(\ell_{2}x+\frac{3\eta \ell_{2}}{\sigma }t\right),\sigma \neq 0$ and $\ell_{1},\ell_{2},\sigma_{1},\sigma_{2},\sigma_{3},\sigma_{4}$ are arbitrary constants. It is seen from Figure 12 that the solution Eq. (21) gives a periodic lump wave, which is going to vanish its wave after a certain times.

## 4 Conclusions

In this paper, we mainly focused the nature of the traveling wave of the (2+1)-dimensional nonlinear CBS model using a dependent variable transformation including a controlling parameter. We explicitly presented the wave interactions such as periodic-soliton, cross-kinky-lump wave, double kinky-lump wave, periodic cross-double kinky-lump wave, periodic two-solitary wave solutions and the breather style of two-solitary wave solutions in analytically as well as graphically. Moreover, we obtained two conditions that made the waves propagated obliquely and orthogonally. Let us point out that the bilinear form of the CBS model and such structural solutions will be useful to investigate many nonlinear dynamics of interaction phenomena in fluids and plasmas fields.

## Declarations

### Author contribution statement

Harun-Or-Roshid: Conceived and designed the experiments; Analyzed and interpreted the data.

Md. Mahbub Hassan: Performed the experiments; Wrote the paper.

Abdul-Majid Wazwaz: Contributed reagents, materials, analysis tools or data.

### Funding statement

This research did not receive any specific grant from funding agencies in the public, commercial, or not-for-profit sectors.

### Competing interest statement

The authors declare no conflict of interest.

### Additional information

No additional information is available for this paper.
